# Recovery and Economic Aspects of Extraction Methods for Artemisinin and its Precursors from *Artemisia annua* L. Leaves

**DOI:** 10.1021/acsomega.5c06842

**Published:** 2025-12-04

**Authors:** Michelle Fernanda Faita Rodrigues, Anna P. Simon, Camila Diedrich, Lucas Vinicius Dallacorte, Bruno Henrique Fontoura, Rubia Cristiani Camochena, Solange Terezinha Carpes, Jorge Freire da Silva Ferreira, José Abramo Marchese

**Affiliations:** † Department of Agronomy, 74354 Federal University of Technology − Paraná (UTFPR), Via do Conhecimento Km 1, Pato Branco 85503-390, Brazil; ‡ Federal Institute of Paraná (IFPR), 2115 Highway BR 163, Zip Code, Barracão 85814-800, Brazil; § Department of Chemistry, Federal University of Technology − Paraná (UTFPR), Via do Conhecimento Km 1, Pato Branco 85503-390, Brazil; ∥ US Salinity Laboratory, Agricultural Water Efficiency and Salinity Research Unit, Riverside, California 92507, United States

## Abstract

A bibliometric analysis of *Artemisia annua* L. research revealed substantial variability in extraction methods and solvents, underscoring the absence of standardized protocols. This methodological inconsistency can lead to differences in extract composition, reducing the reproducibility and comparability of research outcomes. The present study evaluated the recovery and economic aspects of extraction methods and solvents for artemisinin (ART), dihydroartemisinic acid (DHAA), and artemisinic acid (AA) from *A. annua* L. leaves. The extraction methods assessed were — Refluxer (R), Ultra-Turrax Disperser (D), Shaker (S), and Ultrasound Bath (U)— use three solvents: petroleum ether (PE), acetonitrile (ACN), and 70% ethanol (ET). Target compounds were quantified by high-performance liquid chromatography with diode-array detection (HPLC-DAD). ACN was the most efficient in extracting all three compounds, making it the most suitable for analytical and precision-oriented applications. However, ET was identified as a safer, renewable and cost-effective alternative, particularly for ART extraction, and can also enable the coextraction of antioxidant flavonoids with potential pharmaceutical relevance. Among the methods tested, the D technique offered the best balance for laboratory-scale use, combining fast extraction with low solvent consumption and minimal equipment cost.

## Graphical abstract

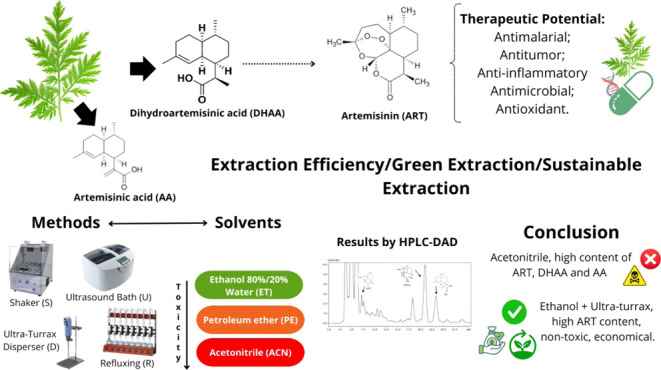

## 1 Introduction

*Artemisia annua* L. (*Artemisia*) gained global attention due to its main bioactive compound artemisinin (ART) and other phytochemicals produced by the plant including dihydroartemisinic acid (DHAA), artemisinic acid (AA), other terpenoids, flavonoids, and coumarins.

The sesquiterpene lactone ART, produced in leaves of Artemisia (0.5–2.0% w/w) is the raw material for ART-based combination therapy (ACT), the first-line therapy in treating malaria where quinine-based drugs are no longer effective.^1–4^ ART-based treatments have been a cornerstone of malaria therapy for several years and are recommended by the World Health Organization (WHO), particularly in cases involving *Plasmodium falciparum* resistance to quinine-based malaria strains.^2^

Beyond its well-known antimalarial properties, *Artemisia* has gained attention in pharmacology, pest control, parasitology, and nutrition, with ongoing research exploring its potential health benefits on animal health. *A. annua* has in vitro activity against parasitic diseases, including schistosomiasis,^5^ Chagas disease, and leishmaniasis,^6^ as well as against viral infections like COVID-19.^7^ More importantly, leaf crude extracts exhibited in vivo bioactivity against cancer,^8^ Hemonchus in sheep,^9^ and an 83.3% cure rate for leishmaniasis in hamsters after 30 days.^10^ Traditional *Artemisia* tea and dried leaves had antimalarial effects in mice.^11^ Additionally, the anti-inflammatory effects of methanolic leaf infusions from *Artemisia argyi* and *Artemisia princeps*—which are not commercial sources of artemisinin—suggest that flavonoids may contribute to the observed bioactivity. Ethanolic extracts of *Artemisia* leaves are rich in antioxidant flavonoids, which have been reported to synergize with artemisinin (ART) against malaria and cancer.^12^ Dried leaves were reported to slow the development of parasite resistance in a malaria mouse model compared to pure artemisinin.^13^

There is growing interest in new applications for *Artemisia*, yet the absence of a sustainable demand for artemisinin and the lack of an optimized extraction method remains as significant barriers to establishing a global artemisinin market. As *Artemisia* remains the most economically viable commercial source of ART, developing efficient extraction techniques is crucial to maximizing yield. These applications hold substantial commercial potential, particularly in Brazil, where the favorable climate supports *Artemisia* cultivation^14–16^ and ET is produced at a large scale for biomass extraction of ART-based and other bioactive products.

Comparatively, ACN and ET extract 6.38 and 14.87 mol of ART, respectively, while toluene at 50 °C yields 130.85 mol.^17^ Despite its low recovery in large-scale processes, 62–70%, hexane is commonly used due to its affordability and effectiveness in extracting epicuticular waxes and oils from leaves, which are precipitated when the dry extract is dissolved with acetonitrile allowing the redissolution of ART, DHAA, and AA.^18,19^

Ethanol was reported to be as effective as hexane and petroleum ether for the extraction and purification of artemisinin^20^ and is a promising alternative for extracting bioactive compounds. The global artemisinin market was valued at USD 64 million in 2021 and is projected to reach USD 217.4 million by 2028, highlighting the need for efficient extraction methods.^21^ Ethanol is renewable,^22^ and approved by the FDA for pharmaceutical applications.^23,24^ Additionally, its polarity, mainly when mixed with water, allows for the coextraction of secondary metabolites, like flavonoids, with potential therapeutic benefits.^1,25^ While other sustainable technologies exist, such as supercritical CO_2_ extraction,^26^ their high costs and specialized equipment requirements limit large-scale use.^27–30^ To prevent interference from waxes and many UV–visible compounds typically extracted with ethanol and methanol, an HPLC-UV quantification method developed by Ferreira and Gonzalez^30^ was validated by ELSD with MS identification of underivatized ART, DHAA, and AA.

We evaluated three solvents previously used for artemisinin extraction with different bulk prices. Petroleum ether, used for the extraction of nonpolar compounds, range in prices, depending on the grade and manufacturer, from 0.5–1.00 USD/L; ethanol (1.0–2.00 USD/L); and acetonitrile (2.00–5.00 USD/L). Acetonitrile HPLC-grade range from 92.00–158.00 USD/L. Artemisinin demand is growing as demand of triple-ART-based antimalarials is aimed at delaying ART-tolerance by *Plasmodium* spp.^31^

The bibliometric analysis conducted in this study revealed substantial methodological variability across the literature regarding extraction techniques and solvents for *Artemisia*. This methodological inconsistency is detrimental, as it directly reduces the reproducibility and comparability of research outcomes, potentially leading to differing extract characteristics and compromised efficacy assessments. Therefore, developing efficient, reproducible, and environmentally responsible extraction protocols is crucial for both industrial and research applications.

The novel contribution of this work is the implementation of a standardized, systematic comparative approach designed to overcome this prevalent methodological inconsistency. Through a 4 × 3 factorial design, our study provides a direct, quantitative benchmark by evaluating the recovery efficiency and economic viability of four distinct extraction techniques—Refluxer (R), Ultra-Turrax Disperser (D), Shaker (S), and Ultrasound Bath (U)—using three solvents selected based on their distinct safety and sustainability profiles: petroleum ether (PE), acetonitrile (ACN), and 70% ethanol (ET). This standardized evaluation is essential for contextualizing existing findings and establishing a reliable, reproducible, low-cost protocol for ART and its precursors that adheres to global pharmaceutical standards.

The primary objective of this study was to evaluate the recovery and affordability of extraction methods and solvents for ART and DHAA extraction from *Artemisia* that are environmentally friendly and align with global pharmaceutical standards.

## 2 Experimental Section

### 2.1 Bibliometric Analysis

A bibliometric analysis was conducted using the Scopus platform to map the scientific research on *Artemisia*, to identify the areas of focus in these studies, and to examine the methodologies employed for artemisinin extraction. The search terms used in the analysis included *A. annua*, *A. annua* L., artemisinin, extraction, quantification, methodology, method, high-performance liquid chromatography (HPLC), and related keywords.

### 2.2 Plant Material and Preprocessing

The *Artemisia* plants, Artemis F2 genotype (MEDIPLANT, Switzerland), were cultivated in the Experimental Area of Federal University of Technology – Paraná (UTFPR), Pato Branco – PR campus (26°07′S and 52°41′W – 760 m of altitude). The leaves were harvested and dried in a forced-air oven at 35 °C for 48 h and then ground using a mill equipped with a 16-mesh USDA sieve (Tecnal, R-TE-650/1), resulting in particle sizes ranging from 0.6 to 1.2 mm. The processed plant material was thoroughly homogenized to ensure consistency and was used for all extraction experiments.

### 2.3 Crude *Artemisia* Extract Preparation

In this study, four different equipments were used to extract ART, DHAA, and AA from plant samples: Refluxer (R), ultraturrax disperser (D), shaker (S), and ultrasound bath extraction (U). The pieces of equipment were manufactured by TE-044–5/50 Tecnal Equipamentos para Laboratorio Ltd.a, IKA T25 Digital ULTRA-TURRAX IKA-Werke GmbH & Co. KG, SL-222 Solab Equipamentos para Laboratórios, and Biofree 06 L Gnatus, respectively. For each method, 0.5 g of oven-dried and ground *Artemisia* leaves was extracted using three different solvents: petroleum ether (PE), acetonitrile (ACN), and 70% aqueous ethanol (ET), prepared with ultrapure water. To account for the potential loss of compounds (extraction recovery), methods that presented the higher ART content were spiked with 1 mg of acetophenone (ACP) as an internal standard to correct for variations in sample preparation, injection, and instrument performance, and to estimate the accuracy and precision of the quantitative analysis. Table 1 summarizes the experimental extraction protocols.

**Table 1:** Summary of Experimental Extraction Protocols of *A. annua* Dried, Ground, Leaves

	crude extraction methodologies			
	refluxer (R)	ultra-turrax disperser (D)	shaker (S)	ultrasound (U)
sample mass	0.5 g Artemisia leaf powder			
extraction solvent	petroleum ether (PE), acetonitrile (ACN), and ethanol 70 vol.% (ET)			
solvent volume (mL)	70	10	70	70
solvent: temperature (°C)	PE: 67(standard method)	25 (PE, ACN and ET)	60 (PE, ACN and ET)	60 (PE, ACN and ET)
	ACN: 82			
	ET 70 vol.%: 79			
extraction process	refluxing using a Goldfish apparatus for 1.0 h. Each solvent used at boiling point	digital disperser extraction for 150 s at 20,000 rpm, 25 °C, followed by a 1.0 h rest	extraction in a shaker/incubator at 150 rpm for 1.0 h	extraction in an ultrasound bath at 35,000 Hz for 1.0 h
HPLC sample preparation	after filtration, the extraction solvent was evaporated to dryness in a hood and reconstituted in 10 mL of acetonitrile using a glass spatula and 30 s of ultrasound. Extracts were filtered (0.45 μm PTFE hydrophilic membrane) and transferred to HPLC vials for chromatographic analysis			

### 2.4 HPLC Analysis

ART, DHAA, and AA were quantified by high-performance liquid chromatography (HPLC, Varian 900-LC, Varian Inc., Walnut Creek, CA, USA) coupled with a diode array detector (DAD) and a C18 reverse-phase column­(250 mm × 4.6 mm i.d., 5 μm) following the method described by Ferreira and Gonzalez.^30^ The injection volume was 10 μL, column temperature 30 °C. The isocratic mobile phase was 60% acetonitrile and 40% 0.1% acetic acid aqueous solution (pH 3.2), flow rate 1.0 mL/min, coupled to a diode array detector set at 195 nm. To determine sample concentrations, a 10-point calibration curve (standards dissolved in acetonitrile) ranged from 0.05 to 0.5 mg/mL. Standards of ART were purchased from Sigma/Aldrich (sigmaaldrich.com) while DHAA and AA were kindly donated by Amyris (Emeryville, CA, https://amyris.com/) through Dr. Jorge Ferreira (USDA-ARS, Jorge Ferreira, US Salinity Lab, Riverside, CA). HPLC-UV was linear in the range tested and had a high linear regression coefficient of *R* ^2^ = 0.9998 between concentrations and peak areas. Results are expressed as ART, DHAA, and AA percent (mg/100 mg plant). Recoveries for ART, DHAA, and AA, when the same sample was extracted by two consecutive hours of refluxing in petroleum ether, were previously reported to range from 95–97% with high intraday and interday repeatability.^30,32–34^

### 2.5 Statistical Analysis

The experiment was conducted using a completely randomized design with a 4 × 3 factorial scheme and three replications. Factor A being extraction methods (Refluxer (R), Ultra-Turrax Disperser (D), Shaker (S), Ultrasound (U)), while factor B was the solvent used (acetonitrile, ethanol, and petroleum ether). Data were analyzed using ANOVA with significance established by the F-test (*p* < 0.05). If a variable was significant, the effects of the extraction method and solvent were compared using the Tukey test (*p* < 0.05). Data analysis and graph construction were carried out using the ExpDes.pt package,^36^ within the RStudio platform.^37,38^

### 2.6 Economic Analysis

An economic analysis was conducted to compare the preparation methods of *Artemisia* crude extracts, specifically refluxer (R), ultraturrax disperser (D), shaker (S), and ultrasound bath extraction (U), using three different organic solvents: petroleum ether (PE), acetonitrile (ACN), and ethanol (ET). The analysis assessed the solvent cost per hour of operation, the cost per sample based on the extraction method and solvent used, and the capital investment required for the extraction equipment.

## 3 Results and Discussion

### 3.1 Bibliometric Analysis

The bibliometric analysis, conducted on the Scopus platform, provided a comprehensive overview of the scientific landscape surrounding *Artemisia* and its bioactive compounds from 1986 to 2024. A total of 226 articles were identified using the specified search terms: *A. annua*, *A. annua* L., artemisinin, quantification, methodology, method, high-performance liquid chromatography (HPLC), extraction, and related keywords. The most frequently occurring keywords in the articles were artemisinin and *A. annua*.

The 50 most cited articles were selected and analyzed individually, with 76% falling within the scope of this research. The remaining articles were excluded due to unavailability, a focus on *Artemisia* tea, discussions of other *Artemisia* species, or topics like nanoencapsulation, artemisinin bioreactor production, and the oral use of artemisinin, all outside the scope of this study.

Upon conducting a more detailed analysis of 39 articles, the bibliometric review revealed the distribution of studies across various research domains. Biotechnology was predominant,^39–47^ comprising 26% of the total, followed by High-performance liquid chromatography methodologies^30,32,48–52^ with 21%. Agronomy^53–59^ and bioactivity applications^5,8,24,60–63^ each accounted for 18%. Phytochemical research^64–68^ represented 13%, while only 5% focused on artemisinin extraction methodology.^18,69^ However, all studies shared a key methodological component: the extraction process, which is a fundamental and integral step to each study, highlighting its critical role in validating scientific investigations across various fields.

Figure 1A illustrates the distribution of several methodologies used for preparing *Artemisia* extracts in laboratory settings. The most employed technique is R, accounting for 29% of the methods used,^32,35,54^ followed closely by agitation at 24%,^19,53,65^ and maceration at 21%.^1,8,17,57^

**Figure 1:**
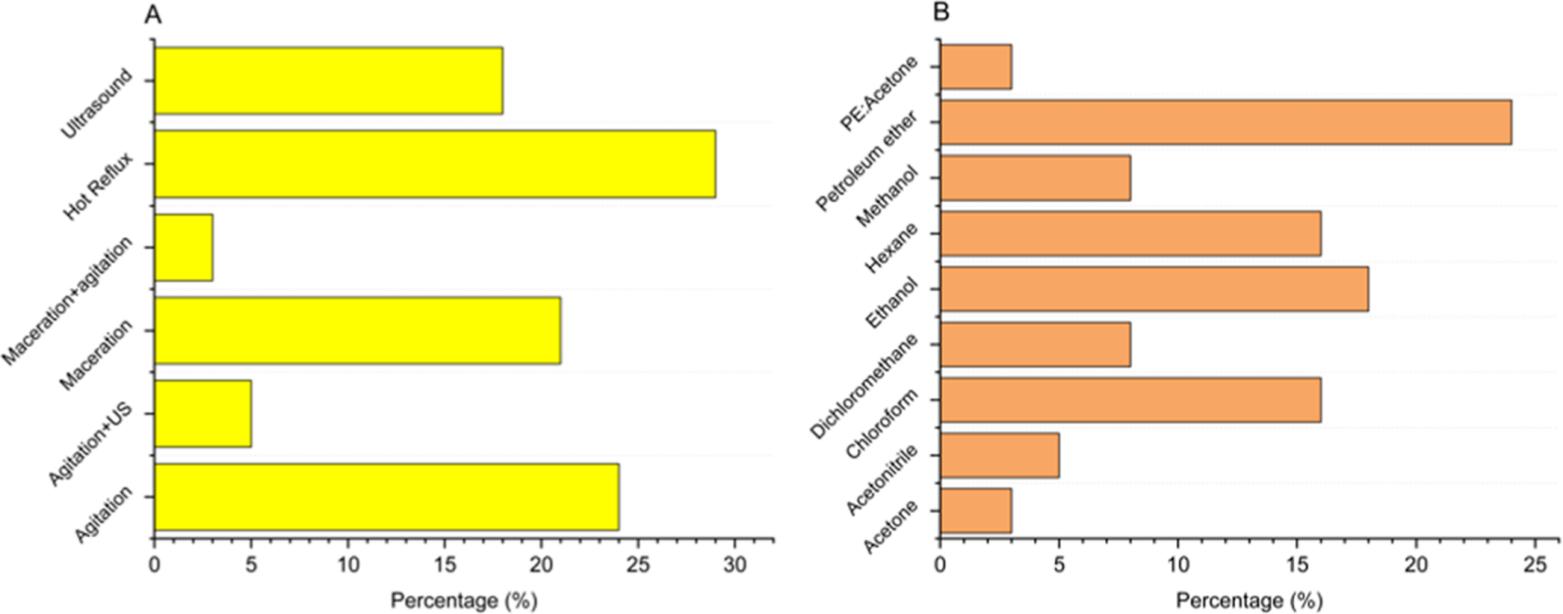
(A) Methodologies utilized to prepare *Artemisia* extracts in laboratory settings based on bibliometric analysis; (B) solvents utilized to prepare *Artemisia* extracts in laboratory settings based on bibliometric analysis.

Among the methodologies reviewed, most studies used PE as the extraction solvent, followed by ET and hexane, as shown in Figure 1B. Extraction with PE is typically done in a Soxhlet apparatus.^39,60,70^ ET is also used for Soxhlet extraction, ultrasound-assisted,^68^ and agitation methods.^5,62^ Artemisinin was also extracted under laboratory settings with PE or other organic solvent using an accelerated solvent extraction (ASE, Dionex/ThermoFisher), pressurized with nitrogen at 1500 psi.^70^ Although more efficient and faster than R, D, and U, the initial costs of the equipment, nitrogen gas, and the stainless-steel cells used for extraction make this method more expensive than the three methods tested here.

Ethers are commonly used for phytoextractions due to their low boiling points and stability to acids, bases, and metals.^71^ For instance, PE’s boiling point ranges from 40 to 60 °C while hexane bp = 69 °C. Also, PE extracted anti-inflammatory compounds from *Haplophyllum tuberculatum* leaves better than chloroform, ethyl acetate, and butanol.^72,73^ While ethanol and methanol are not effective for extracting polysaccharides, tannins, gums, and waxes, they are excellent solvents for alkaloids, flavonoids, terpenes, and glycosides.^74^

The bibliometric analysis revealed a significant variation in methods and solvents used, highlighting the lack of a standardized approach for *Artemisia* extracts. This diversity in techniques suggests that different studies may produce extracts with distinct characteristics, potentially leading to inconsistencies in research efficacy and reproducibility. Such variability underscores the urgent need to develop and adopt standardized extraction protocols. Standardization of extraction would promote consistency across studies, enhancing the reliability and comparability of findings in *Artemisia* research, crucial for studies on bioactive compounds and their potential applications.

### 3.2 Quantification of Artemisinin, Dihydroartemisinic Acid, and Artemisinic Acid in Crude Extract

The comprehensive comparison of the four different extraction methods combined with three extraction solvents used for ART, DHAA, and AA extraction revealed distinct trends, as shown in Figure 2A–C. As expected, the medium-polarity solvent ACN consistently demonstrated superior extraction recovery for all three compounds across all extraction methods, being ∼1.1–1.2, 0.2–0.45 and 0.012–0.030% (g/g dry leaves). This can be attributed to ACN moderate polarity and aprotic nature, that enhance solubility across a broad range of metabolites, making it particularly effective for achieving precise and reproducible quantification in laboratory-scale studies. For ART extraction, 70% ET was the most effective with the D method, indicating that ART is unaffected by 30% ultrapure water. There was no difference for PE recovery across all methods.

**Figure 2:**
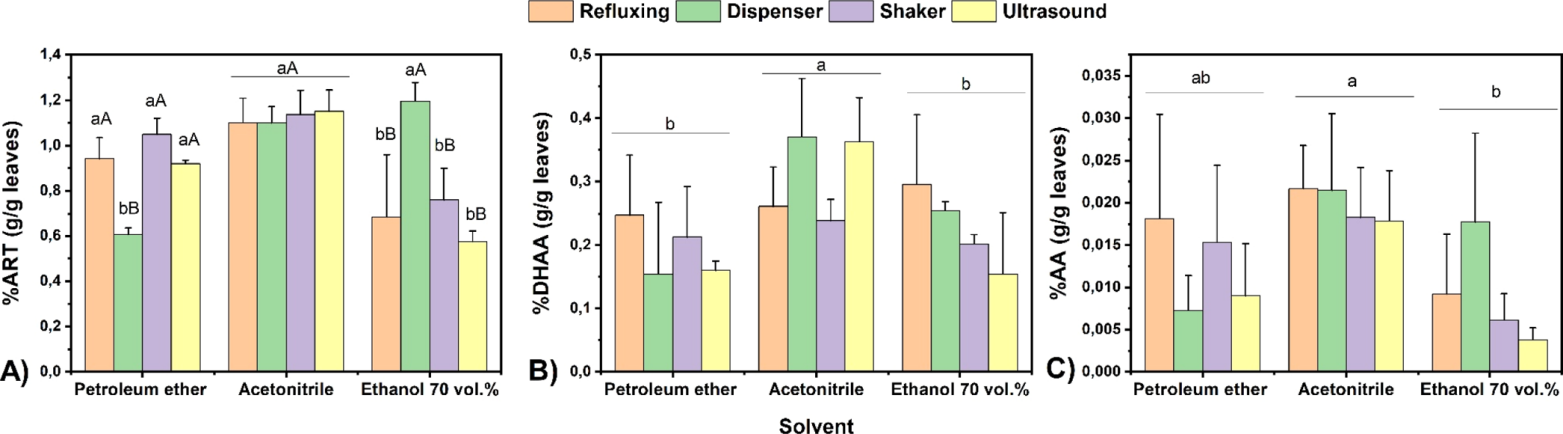
Comparison of (A) Artemisinin (ART), (B) dihydroartemisinic acid (DHAA), and (C) artemisinic acid (AA) extraction percentage using different extraction methods/equipments and solvents. Note: Uppercase letters A–B compare the solvents between all the extraction methods, while lowercase letters a-b compare the solvents within each extraction method. Both comparisons are based on Tukey’s test, and values that share the same letter do not exhibit statistically significant differences.

In the case of DHAA extraction (Figure 2B), occurring in concentrations lower than ART in leaves, the high coefficient of variation (CV) of 31% suggests considerable variability among methods. However, ACN provided the best extraction recovery for ART, DHAA, and AA of all solvents (Figure 2A–C). The higher variability observed for DHAA can be attributed to its structural complexity, making it more challenging to achieve reproducible extractions across the different methods.

The findings suggest that ACN is the most effective solvent for extracting ART, DHAA, and AA, making it the preferred choice for extracting ART and other metabolites from *Artemisia* plant material for content determination. However, ET did not show a statistically significant difference in ART extraction when combined with the disperser method. ET is recognized as one of the safest solvents for extracting bioactive compounds from plants, including essential oils, FDA.^24,75^

ACP recovery percentage were 97% and 98% for ACN and ET extraction when using the Dispenser method, while for Refluxing using petroleum ether were 98% (standard methodology). These values are in accordance with Ferreira and Gonzalez.^30^

The systematic evaluation of extraction efficiency conducted in this study (ART concentrations ranging from 0.38% to 1.25%) establishes a robust, standardized framework for comparing extraction yields across solvents of varying polarity. By aligning our results with previous reports, this approach enhances methodological consistency and supports the reproducibility of *Artemisia* extraction studies.

Our findings confirm that 70% ET achieves high recovery rates for ART and its biosynthetic precursors, representing an efficient and economically feasible alternative to more polar solvents. This observation is consistent with the literature, where crude ethanolic extracts (70% aqueous ET) have shown comparable quantification results — with a Brazilian cultivar yielding 1.26% ART and a Chinese cultivar 2.72% ART, along with 0.9% and 1.5% DHAA, respectively.^5^ Similarly, studies optimizing hydroalcoholic extraction have demonstrated a positive correlation between ET concentration and ART content, increasing from 2.0 mg/mL in 25% ET to 7.9 mg/mL in 90% ET extracts.^62^ On a preparative scale, ET (96° GL) has been employed successfully for artemisinin isolation, achieving global process recoveries of approximately 73%.^25,83^ Collectively, these results reinforce ethanol’s suitability as a renewable, safe, and efficient solvent for both analytical and preparative extractions.

In contrast, PE remains a solvent of choice in analytical and industrial applications due to its nonpolar selectivity, which yields cleaner chromatographic profiles. In our study, the refluxing procedure with PE achieved high recovery efficiencies (approximately 98% for DHAA, ART, and AA), aligning with previously reported values ranging from 95% to 97% using two consecutive hours of refluxing.^32^ Moreover, PE applied in Accelerated Solvent Extraction (ASE) systems achieved extract concentrations up to 1.15% ART in leaves, while conventional refluxing for 60 min yielded approximately 70% of the artemisinin obtained by pressurized liquid extraction.^69^

Acetonitrile (ACN), however, demonstrated the highest extraction recovery for ART, DHAA, and AA across all evaluated methods, establishing it as the optimal solvent for analytical determination. Its superior performance aligns with reports indicating that maceration with ACN (24 h shaking in the dark) extracts over 90% of the artemisinin present in herbal material.^52^

The HPLC results (Figure 3) demonstrate that the solvent had a significant impact on the chromatographic profile, affecting the polarity and quality of the extracted compounds as well as the interpretability of HPLC-DAD results. The retention times for ART, DHAA, and AA were 7.44, 14.65, and 16.44 min, respectively. ART concentrations varied between 0.38% and 1.25% across the different extraction methods and solvents.

**Figure 3:**
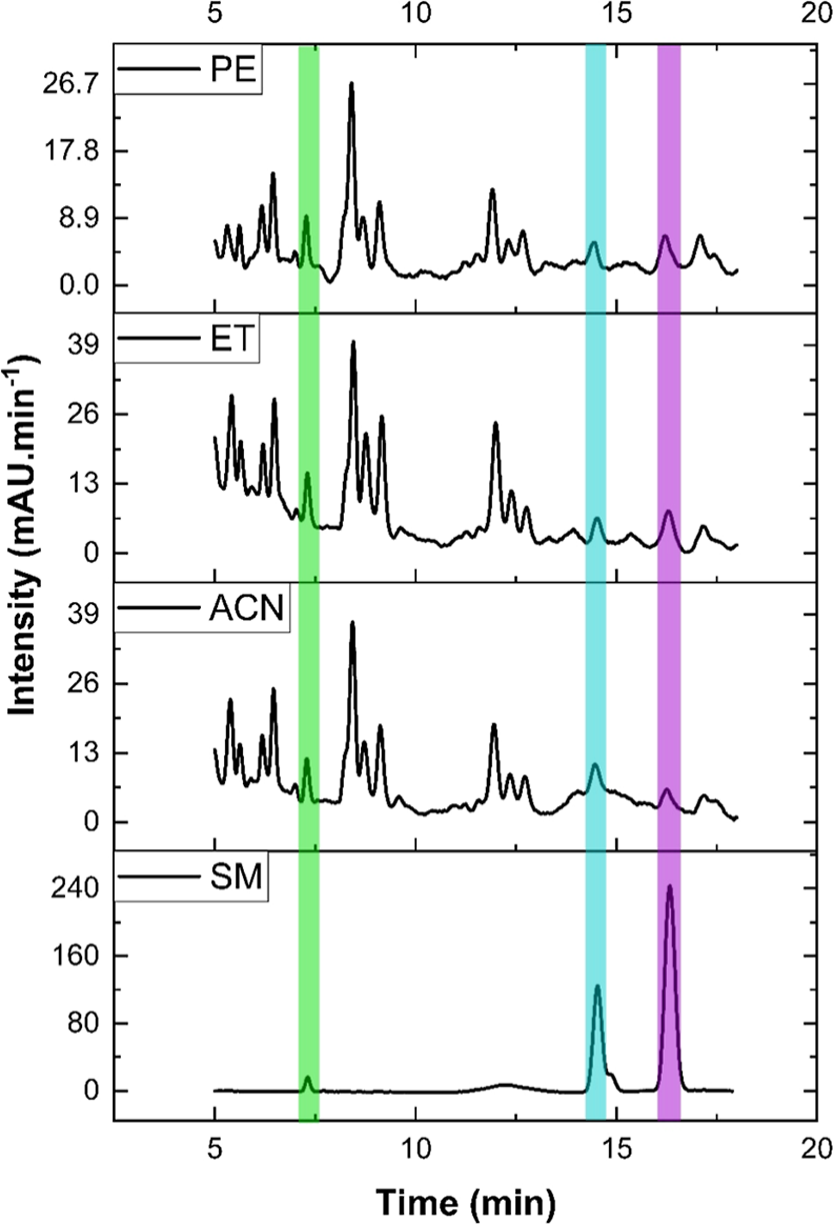
HPLC-DAD chromatograms of a standard mixture (SM) of artemisinin (ART-green), dihydroartemisinic acid (DHAA-blue), and artemisinic acid (AA-purple) at 0.25 mg·mL^–1^ in ACN, and of *Artemisia* extracts obtained by Ultra-Turrax Disperser methodology using petroleum ether (PE), acetonitrile (ACN), and aqueous ethanol at 70% (ET).

The choice of solvent significantly impacted the extraction recovery of ART and its related compounds. Protic polar solvents like ET can effectively extract ART and several secondary metabolites, as indicated by early retention peaks in the HPLC chromatograms (Figure 3). ET-based extracts are particularly rich in phenolics and flavonoids, which possess antioxidant and immunomodulatory properties.^76–79^ These compounds may synergize with ART, potentially enhancing its therapeutic efficacy and improving the effectiveness of ART-based treatments.^12^

Medium-polarity aprotic solvents like ACN are widely used in plant extract preparation due to their ability to solubilize both polar and moderately polar compounds, resulting in a well-balanced extraction profile.^80^ *Artemisia* extracts obtained with ACN show similarities to those extracted with 70% ET (Figure 3) yielding comparable concentrations of ART and its metabolites. Besides providing high recovery rates, ACN is miscible in water and, when combined with an acidic aqueous phase as a mobile phase, it removes ART and its two related acids from the HPLC column.^30^

Nonpolar solvents like PE, on the other hand, selectively extract nonpolar compounds, such as sesquiterpenes, oils, and waxes, later precipitated by the ACN extraction of the dry *Artemisia* PE extract, resulting in a chromatogram with fewer interfering UV–visible compounds and enriched in the target sesquiterpene compounds.^30,77^ This cleaner extraction profile helps minimize baseline noise, coelution issues, and other disturbances, ensuring more accurate quantification of ART, DHAA, and AA.^30,77^ Phenolics and antioxidants commonly extracted with ART in ET possess immunomodulatory properties that may enhance ACT’s antimalarial efficacy.^25,64^ These synergistic interactions can provide broader therapeutic benefits, making the inclusion of coextracted secondary metabolites a promising strategy for optimizing ART formulations^15^ with potential to delay tolerance to ACTs.

Both flavonoids and sesquiterpenes contribute to a wide range of biological effects, including antioxidant, anti-inflammatory, antimicrobial, anticancer, and neuroprotective activities.^61,65,76,80,81^ In antimalarial formulations, several studies suggest that these coextracted compounds may enhance the efficacy of ART through synergistic pharmacological mechanisms. Phenolics and antioxidants commonly extracted from Artemisia using ET possess immunomodulatory properties that may enhance their therapeutic efficacy in malaria treatment.^25,64^ These synergistic interactions can provide broader therapeutic benefits, making the inclusion of coextracted secondary metabolites a promising strategy for optimizing artemisinin formulations and improving ART-based therapies.^32,83,84^ Additionally, such synergistic interactions are believed to contribute to broader therapeutic benefits, making the inclusion of coextracted secondary metabolites a promising strategy to delay tolerance to ART-based formulations.^15^

### 3.3 Economic Analysis

The economic evaluation of extraction methodologies and solvents for *Artemisia* highlights key factors influencing operational costs, cost per sample, and capital investment. The selection of an optimal solvent must consider not only its extraction recovery but also physicochemical properties such as solvent power, boiling point, reactivity, viscosity, recovery rate, vapor pressure, safety, toxicity, cost, and its applicability in large-scale processes.^74^

Recent studies showed that even laboratory-scale experiments can predict large-scale extraction costs on a theoretical basis.^26,84,85^ The findings suggest that solvent type plays a critical role in determining the total operational expenditure associated with artemisinin extraction, isolation, and purification. *Artemisia* extraction recovery for high-purity artemisinin can reach 72% at the lab scale.^84^

The cost per hour of operation varied significantly across the tested solvents. We found that ET was the most economical solvent (Figure 4A) due to its renewable nature and widespread production in Brazil.^26,85^ In contrast, petroleum ether (PE) is the most frequently used solvent for *artemisinin* extraction (Figure 1B), despite its higher operational costs when employed in shaker-based methods. PE’s low polarity and boiling point (40–60 °C) facilitate the removal of nonpolar impurities such as waxes and lipids, contributing to its widespread adoption. However, its cost-effectiveness must be weighed against its moderate safety profile (NFPA rating of 1).^18,19,32,85^

Petroleum ether is the most used solvent for *artemisinin* laboratory extraction, as indicated by the bibliometric analysis in Figure 1B. However, it incurs the second highest cost per hour of operation (Figure 4A), particularly when using shakers. This preference for PE may be attributed to its relatively low health risk to the operator, scoring a 1 on the National Fire Protection Association (NFPA) scale (New Environment Inc. - NFPA Chemicals, n.d.), while ET and ACN score 2 and 3, respectively. Additionally, PE’s lower boiling point (40–60 °C) compared to ACN (82 °C) and ET (78 °C) enables faster processing at lower temperatures, advantageous for refluxing - the most used extraction method (Figure 1A). Furthermore, PE is favored because it has low polarity and is effective in removing greases and waxes (impurities) from plant material.^18,19,32,85^ However, it costs more than hexane, which extraction efficiency are around 70–75% with a final yield of 40–65% after purification.^86^

**Figure 4:**
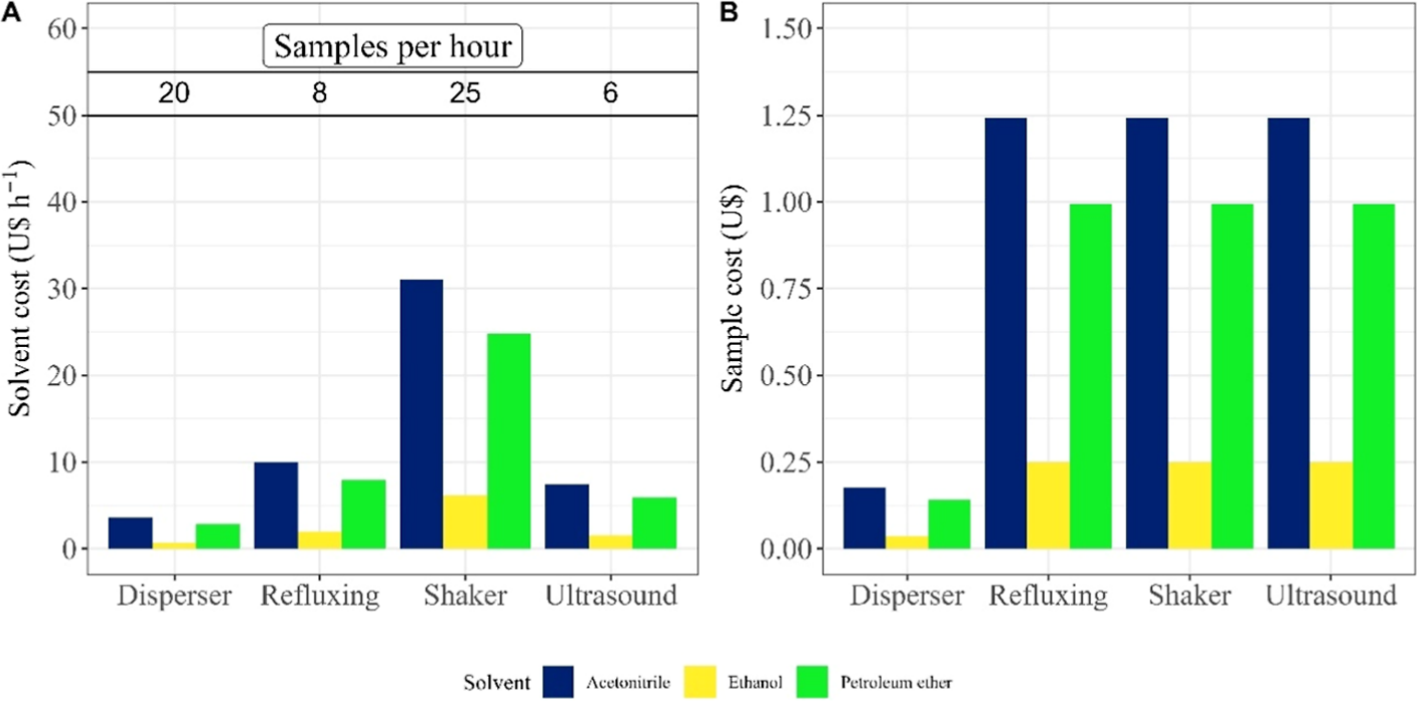
(A) Solvent cost and sample throughput per hour of operation. (B) Sample cost for the tested extraction methodologies.

Acetonitrile (ACN), despite its superior extraction recovery and chromatographic suitability, was the most expensive solvent tested (Figure 4A). Its aprotic nature and moderate polarity enable the solubilization of a broad spectrum of compounds, making it an essential solvent in high-performance liquid chromatography (HPLC) and liquid–liquid extraction (LLE) applications.^52^ The high miscibility of ACN with water facilitates efficient phase separation, while its low viscosity enhances chromatographic resolution and detection sensitivity. However, its elevated cost and safety considerations (NFPA rating of 3) precludes its feasibility in large-scale extractions.^82^

Thus, considering extraction recovery, costs, safety, and applicability, ET was identified as the most suitable solvent for artemisinin extraction.^84,86^ Recognized as a safe solvent by the FDA,^23,24^ ET also presents additional advantages, such as bacteriostatic properties, scalability, renewable and cost-effectiveness. These characteristics make it the most economical and feasible solvent among those evaluated (Figure 4).

Despite the widespread perception of ET as a renewable and environmentally benign solvent, few studies have applied a formal Life Cycle Assessment (LCA) to quantitatively substantiate its environmental benefits. Sustainability claims for ET are often based on its renewable origin or biodegradability, rather than on comprehensive cradle-to-grave evaluations. As highlighted by Roberto Ometto et al.,^87^ the environmental performance of biobased ET depends critically on methodological decisions such as system boundaries, allocation procedures, and inclusion of agricultural inputs. Without standardized LCA frameworks, these factors are frequently handled inconsistently, resulting in variable and occasionally overstated “green” claims.

Formal and detailed LCA conducted for sugar cane ethanol^87^ in Brazil demonstrated substantial greenhouse gas (GHG) reductions relative to fossil fuels, largely due to renewable energy use from bagasse and efficient agricultural management. Nevertheless, both studies emphasize that these advantages are context-dependent and sensitive to regional production practices, energy sources, and land-use dynamics. The absence of consistent LCA evaluations therefore weakens the quantitative foundation for ET’s environmental superiority and underscores the need for transparent, data-driven analyses.

When compared to petroleum-derived solvents such as PE, ET shows a markedly lower fossil carbon footprint. PE production relies on energy-intensive distillation of nonrenewable crude oil, contributing to elevated GHG emissions and high cumulative energy demand.^86,87^ In contrast, ethanol—particularly from sugar cane—comes from a renewable carbon cycle in which much of the CO_2_ released during use is reabsorbed during feedstock growth.

Our evaluation of extraction methodologies revealed substantial differences in operational costs (Figure 4A). The shaker exhibited the highest cost due to its large sample processing capacity, followed by the refluxer, ultrasound, and dispenser. In terms of unit cost per sample (Figure 4B), the D method demonstrated the lowest cost due to its minimal solvent requirement, making it highly efficient for both research and industrial applications.

Investment acquisition analysis (Table 2) identified the U method as the most cost-effective in terms of equipment expenditure but with low throughput. The D provided an optimal balance between cost and throughput, processing up to 20 samples per hour. The S, while associated with higher operational expenses, offered greater processing capacity, making it suitable for high-throughput research environments. Conversely, the R required the highest capital investment yet exhibited relatively low processing capacity, which may constrain its cost-effectiveness in routine extraction workflows. Other authors have discussed the Soxhlet extraction system as suitable for large-scale extractions but is not considered green.^71,74^

**Table 2:** Estimated Investment in Equipment Acquisition

equipment	equipment cost (USD)
refluxer (R)	3191.50^T^ ^a^
disperser (D)	1773.00^F^ ^b^
shaker (S)	2659.50^N^ ^c^
ultrasound (U)	620.50^P^ ^d^

a ^T^Tecnal – Piracicaba, Brazil.

b ^F^Fortlab – Salvador, Brazil.

c ^N^Newlab – Piracicaba, Brazil.

d ^P^Prolab – São Paulo, Brazil. Accessed in July 2024.

The Ultrasound was incorporated into factorial design to assess its extraction efficiency and economic feasibility. However, this study did not include a systematic evaluation of key ultrasonic parameters—such as frequency, power density, and duty cycle—due to instrumental constraints. The available equipment consisted of a conventional ultrasound bath unit (Biofree 06 L, Gnatus) operating at a fixed frequency of 35 kHz for 1 h, which limited precise control over acoustic intensity and frequency modulation. The selection of this apparatus was guided by its accessibility and low capital cost (USD 620.50). Comprehensive optimization of ultrasonic extraction typically requires high-power probe-type systems capable of parameter modulation, which were not available for the present study and entail substantially greater investment.

## 4 Conclusion

The substantial methodological variability identified in the literature on *artemisinin* extraction underscores the need for standardized and reproducible protocols to enhance the reliability and consistency of research outcomes. The present study provides a data-driven, comparative assessment of extraction techniques and solvents, addressing this critical gap.

Our systematic evaluation demonstrated that acetonitrile (ACN) was the most effective solvent for quantifying artemisinin (ART), dihydroartemisinic acid (DHAA), and artemisinic acid (AA) across all methods tested. However, it shows high cost and safety limitations. In contrast, 70% ethanol (ET) emerged as the most practical, renewable, and cost-efficient alternative. ET not only enabled high ART recovery but also ensured safety and compliance with pharmaceutical-grade solvent standards. Furthermore, its use promotes the coextraction of bioactive compounds—such as flavonoids and phenolics—that may exert synergistic effects in artemisinin-based therapies.

Among the evaluated techniques, the Ultra-Turrax disperser (D) method proved to be the most suitable for laboratory-scale applications. This approach provided an optimal balance between extraction efficiency and operational simplicity, requiring minimal solvent volume and modest equipment investment. Overall, the standardized comparative framework developed in this study establishes a reliable methodological foundation for future research on *Artemisia* extraction, supporting the development of efficient and environmentally responsible laboratory protocols.

The identification of 70% ET as the most economical and environmentally preferable solvent underscores the need for quantitative validation of its sustainability profile.

Additionally, the current limitation in ultrasonic equipment highlights opportunities for optimizing extraction parameters such as power, frequency, and energy density to enhance efficiency and energy performance. The coextraction of flavonoids and phenolics by ET also presents a promising avenue for biomedical exploration, as these compounds may act synergistically with artemisinin to improve therapeutic outcomes and support the development of more effective plant-based pharmaceutical formulations.
